# Grounding cognition: heterarchical control mechanisms in biology

**DOI:** 10.1098/rstb.2019.0751

**Published:** 2021-03-15

**Authors:** William Bechtel, Leonardo Bich

**Affiliations:** ^1^ Department of Philosophy, University of California San Diego, La Jolla, CA, USA; ^2^ IAS-Research Centre for Life, Mind and Society, Department of Philosophy, University of the Basque Country (UPV/EHU), Avenida de Tolosa 70, Donostia-San Sebastian 20018, Spain

**Keywords:** decision-making, production mechanisms, control mechanisms, chemotaxis, circadian rhythms

## Abstract

We advance an account that grounds cognition, specifically decision-making, in an activity all organisms as autonomous systems must perform to keep themselves viable—controlling their production mechanisms. Production mechanisms, as we characterize them, perform activities such as procuring resources from their environment, putting these resources to use to construct and repair the organism's body and moving through the environment. Given the variable nature of the environment and the continual degradation of the organism, these production mechanisms must be regulated by control mechanisms that select when a production is required and how it should be carried out. To operate on production mechanisms, control mechanisms need to procure information through measurement processes and evaluate possible actions. They are making decisions. In all organisms, these decisions are made by multiple different control mechanisms that are organized not hierarchically but heterarchically. In many cases, they employ internal models of features of the environment with which the organism must deal. Cognition, in the form of decision-making, is thus fundamental to living systems which must control their production mechanisms.

This article is part of the theme issue ‘Basal cognition: conceptual tools and the view from the single cell’.

## 1. Introduction

The term *cognition* applies to diverse phenomena that figure in adaptive interactions between biological organisms and their environments. There is no agreement on whether and how it is possible to trace the boundaries between cognitive and non-cognitive (biological only) activities of biological systems. Are unicellular systems that are capable of chemotaxis, communication, vision already ‘cognitive’ [1]? Or is a nervous system required, with the consequence that cognition is only found in animals [2]? Or is it restricted to organisms with a neocortex, or even to humans [3]? How one answers these questions has implications ranging from how one characterizes the core mechanisms involved in cognition and the relationship (hierarchical or heterarchical) between them, to how one selects model organisms for studying cognition.

This paper joins the debate on the side that cognition is a fundamental activity in all living organisms; Jennings [4] and Washburn [5] were early advocates for adopting an inclusive view of cognition, defended more recently by Maturana & Varela [6] and Lyon [7], and adopted by many of the papers in this theme issue. Our particular contribution focuses on one cognitive activity, decision-making, which we argue is performed by the control processes that are required for organisms to be autonomous. All living organisms are autonomous in that they belong to lineages of organisms that maintain themselves as distinct, organized systems far from equilibrium with their environment despite natural processes that would lead to their degradation [8]. To do so, they must procure matter and energy from their environment and use these to construct and repair themselves. They carry out these activities of construction and repair using a variety of mechanisms that perform physiological and behavioural activities. These basic mechanisms, which we refer to as *production mechanisms* [9,10], extract and use energy, break down and synthesize materials, move organisms through space, enable division or replication of the organism, etc. The successful operation of these mechanisms is required since otherwise the organism would not be able to maintain its identity distinct from its environment. However, most organisms encounter variable environments, both internally and externally. Accordingly, organisms must adjust the operation of their production mechanisms so that they perform operations needed to maintain the organism at that time. They require *control mechanisms* that regulate the operation of production mechanisms and make decisions about when to use production mechanisms [9,10]^1^.

Keijzer [12], adopting a ‘radical embodied view’ on cognition, challenges the claim that making decisions is sufficient for cognition; he maintains that full-bodied biological agency, generating actions in the world, is required. While we agree with Keijzer in situating cognition within agents, we maintain that what makes activity cognitive is what the agent contributes. In decision-making, the agent selects which production mechanisms to deploy based on applying norms to measurements.

To begin, we offer a very simple example to illustrate the basics of our account of decision-making in the context of a biological autonomous system, focusing on just one component—an allosteric enzyme. An allosteric enzyme has two binding sites: a sensory site at which binding a molecule alters the enzyme's conformation and an effector site that is a production mechanism that catalyses a reaction. On our view, the allosteric enzyme makes a decision as it selects, based on the interaction with a molecule at the sensory site, how the effector site functions. This decision determines the rate of the reaction. Moreover, the allosteric enzyme does so in accord with a norm that is realized in the constitution of the allosteric enzyme itself. This norm is not something represented or selected by the enzyme itself, but incorporated into it. The reason to identify the constitution of the allosteric enzyme as embodying a norm is that it determines how the enzyme will act. Moreover, calling it a norm is not just a gloss offered by a theorist—it is established in the enzyme by the organism. Like all mechanisms in an organism, an allosteric enzyme is dependent on the organism for its construction and repair, and for providing it with the matter and energy it needs to perform its activities. In turn, by its control operations, an allosteric enzyme contributes to the maintenance of the biological system that harbours it. The control mechanism is not an external imposition on the production mechanism, but an integral part of the organism itself. Through its actions it is making decisions *for* the organism. Selecting the kinetics of a reaction is a minimal example of making a decision; below we develop examples where the selection is between different production mechanisms. But this example suffices to illustrate our characterizations of decisions as requiring making *measurements* and applying *norms* to make *selections*.

As the framework of production and control mechanisms is fundamental to the account that we are advancing for understanding cognition, we develop it further in §2. In §3, we analyse some biological examples of production and control mechanisms, and in §4, we focus on how control mechanisms are involved in cognitive activities such as decision-making. In §5, we turn to what is an important feature of control mechanisms in living organisms, how they are organized.

In both machines and human institutions, control mechanisms are often organized hierarchically [13,14]. In a hierarchy, individual control mechanisms are themselves controlled by a higher-level control mechanism, with a single controller ultimately in charge. The system is organized as a pyramid. In living systems, however, control mechanisms are typically organized heterarchically. We use the term *heterarchy*, first introduced by McCulloch [15], for major deviations from hierarchy such as when a given production mechanism is regulated by multiple control mechanisms without these control mechanisms being themselves subsumed under a higher-level controller. To the degree one can distinguish levels of control, there may be more controllers at higher levels than at lower levels [9]. As we construe these control mechanisms as effecting decisions, on our account decision-making is highly distributed and heterarchical.

Throughout we will illustrate decision-making in bacteria and other organisms in which norms embodied in a decision-making mechanism are applied directly to measurements made by the control mechanism. But a more complex form of decision-making arises when organisms rely on internal models of the world (the concept of internal model is developed in control theory, especially as applied to neural control of motor activity; see [16–18]). This may seem to go far beyond the capacities of bacteria, but in §6 we discuss how some species of cyanobacteria rely on an internal representation of the light–dark cycle (a circadian clock) to regulate a host of activities. In a brief conclusion, we draw out the lessons that are learned by understanding decision-making in the context of control mechanism and so grounding cognition in an important demand placed on all organisms.

## 2. Production and control mechanisms

To explain a phenomenon, biologists typically advance an account of the responsible mechanism. The new mechanists in philosophy of science have focused on this practice and offered accounts of what mechanisms are [19–21] and how they are discovered [22,23]. On these accounts, mechanisms generate phenomena as a result of the coordinated activities performed by their constituent parts—individual parts perform activities that generate conditions for other parts to perform their operations. These accounts have not distinguished between mechanisms that produce materials or activities of the organism and those that exercise control over other mechanisms.

An alternative framing of what a mechanism is will be useful for distinguishing production and control mechanisms and understanding the role of control mechanisms in cognitive phenomena such as decision-making: *mechanisms are systems of constraints that restrict the flow of free energy to perform work* [24]. This is illustrated in a steam engine: steam is constrained to flow and apply force to the components of attached machines. The notion of constraint derives from classical mechanics [25], which confronted the challenge of explaining the behaviour of macroscale objects in terms of Newton's force laws. Constraints stand in an asymmetrical relationship to dynamics as they impose boundary conditions on dynamical relations. Newton's Laws allow for movement of particles in any of six degrees of freedom. Constraints restrict what movements are possible. However, as Hooker [26] makes clear, constraints also open up possibilities—water constrained by a pipe can reach a destination much further away than if it is not so constrained.

Biological systems rely for their activities on constraints they produce. To maintain themselves as organized systems in far from equilibrium conditions—i.e. in highly improbable dynamic distributions of molecules and supramolecular structures—organisms need to recruit and exploit the thermodynamic flow by means of structures that act as constraints, i.e. as local boundary conditions that enable specific processes which can be used to perform some coherent activity in the context of the system [27,28]. The constraints are locally unaffected by the processes they enable. An enzyme is an example in which constraints lower the activation energy necessary for a reaction, thereby catalysing the production of an otherwise improbable product, while not being consumed in the reaction. At a different scale, the vascular system operates similarly to pipes. It canalizes the distribution of blood towards specific organs, which could not be accomplished by diffusion alone. As a result of constraints, production mechanisms function to synthesize, repair and replace components of the organism and generate its basic activities and behaviours. What is distinctive of biological organisms is that these constraints and the mechanisms they constitute collectively contribute to maintaining the conditions for continued existence, thus realizing a causal regime called ‘closure of constraints' [29]. In this view, living systems are autonomous; they maintain themselves and self-maintenance is their ultimate norm.

To be effective in maintaining the organism, production mechanisms must be controlled so that they operate when and how they are needed, not whenever their start-up conditions (i.e. energy requirements and presence of material substrates) are met. Uncontrolled operation is impossible: owing to energetic and spatial limits, cells simply cannot synthesize all the possible proteins and other molecules at the same time. Control must be exercised over biosynthetic processes in order to produce the necessary components at the right times and as needed given external and internal conditions facing the organism. In addition, different subsystems might present different ways of operating, and their activities and rates need to be coordinated to avoid conflict and to ensure their joint functional contribution to the maintenance of the system.

Let us consider, for example, the different and competing production mechanisms involved in the metabolism of glucose and glycogen in mammals (glucose intake, glycolysis, glycogenolysis, gluconeogenesis, glucose transport, etc.), which need to be coordinated by hormones such as insulin and glucagon released by the pancreas [30]. The problem of avoiding conflict and coordinating production mechanisms with competing requirements is common to all biological systems. This is also acknowledged as a major issue in the debate on the origins of life [31,32], where one of the main challenges is to understand specifically how metabolism, membranes and genetic components could come together and realize concerted operations [33].

To functionally modulate the operations of production mechanisms, all biological systems rely on a specialized class of constraints that realize the second-order mechanisms that control production mechanisms in relation to specific variations induced by both environmental perturbations and the internal dynamics of the organism [9,11,27]. To be controlled, production mechanisms must have flexible or variable constraints. Such constraints do not permanently reduce the degrees of freedom—as the flexible constraint is changed, so are the degrees of freedom left open in the controlled mechanisms. By operating on these flexible constraints, control mechanisms enable the organism to act on and change its own dynamical behaviour. Control mechanisms are also flexible insofar as they are sensitive to specific features of their medium and change their state accordingly. Allosteric enzymes, as we described above, show how this is possible. The binding of a ligand to the sensory site results in a conformation change, altering soft constraints in the effector site and thereby its ability to catalyse a reaction. The operations of a control mechanism in turn can be modulated by other control mechanisms that operate on flexible constraints in the first control mechanism. Multiple control mechanisms can be assembled into an architecture capable of determining multiple different specific responses as needed to maintain the complex biological organization constituting an organism.

## 3. Biological examples of control and production mechanisms

To make this discussion concrete, we consider two biological examples of production mechanisms in bacteria—protein synthesis and motility with a flagellum. Protein synthesis involves first the transcription of DNA into mRNA, then the translation of mRNA into peptide chains, and finally the folding of the peptide chains. Each step requires production mechanisms. Transcription is constrained by the enzyme RNA polymerase, which binds to the promoter sequence on the DNA, separates the two strands, and produces a strand of mRNA by adding RNA nucleotides. After the bonds between DNA and RNA molecules are broken, the mRNA strand is further modified (e.g. polyadenylation, capping and splicing) by other enzymes [34]. Translation of mRNA into a peptide chain occurs at the ribosome, where the mRNA strand provides a template. As the mRNA moves through the ribosome, one codon at a time, it becomes the target to which a tRNA molecule ferries a corresponding amino acid. There the amino acid is added to the developing polypeptide chain [34]. In the translation step, the ribosome, the mRNA and the tRNA are constraints [35]. The structure of the protein is then further constrained into a three-dimensional structure by chaperone enzymes to achieve functionality and contribute to the maintenance of the system that produced it.

These production mechanisms—the DNA and RNA polymerase, the mRNA, tRNA and ribosomes, and the chaperones, must be controlled so that the appropriate proteins are produced when they are needed. This is accomplished through, for example, repressor or activator proteins, control mechanisms that bind to DNA. Repressors block the RNA polymerase from gaining access to the promoter site of a gene or an operon (a set of genes) unless an appropriate molecule binds to the sensory site, causing a conformation change that makes the repressor no longer able to bind to the promoter site, thereby allowing transcription to commence.

**Figure 1.**
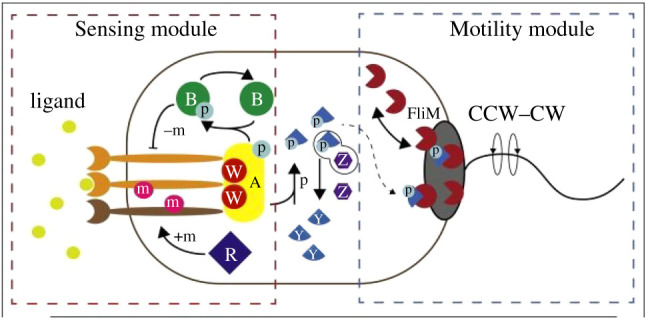
The Sensing and Motility modules involved in chemotaxis in *E. coli*. p, phosphate; m, methyl group; B, CheB; R, CheR; W, CheW; Y, CheY; Z, CheZ; CW, clockwise; CCW, counterclockwise. From *Current Opinion in Microbiology,* Micali & Endres [42], Fig. 1, © 2016, reproduced with permission from Elsevier. (Online version in colour.)

Our second example is bacterial chemotaxis. Bacteria such as *Escherichia coli* use flagella to swim. Chemotaxis enables them to control swimming in response to changing gradients of metabolites or toxins in the environment [36–41]. In this case, the *production mechanism* is the flagellum, a complex molecular mechanism consisting of a filament rotated by a motor (this is the Motility module shown on the right side of figure 1) [43]. If the motors on the five to ten flagella of an *E. coli* all rotate in a counterclockwise direction, the filaments form a coherent structure that propels the bacterium forward (running behaviour). If one or more motors rotates clockwise, the filaments separate, generating no propulsion and the bacterium tumbles. Normally, the motors switch between the two directions, resulting in the bacterium moving in one direction, tumbling, and then moving in another random direction. By controlling the motors, the bacterium is able to bias the switching—continuing counterclockwise rotation and movement in a given direction for a longer period before switching, which is desirable when the bacterium is climbing a metabolite gradient or moving down a toxin gradient. FliM molecules at the base of the motors are flexible constraints—when phosphorylated CheY binds to FliM, a given motor rotates clockwise [44–46].

To decide how to move, bacteria use transmembrane methyl-accepting chemotaxis proteins (MCPs) to measure metabolite or toxin gradients [38,39,47]. *E. coli* has five types of MCP, identified by the ligands to which they enable responses—Tsr (to serine and repellents), Tar (to aspartate and repellents), Tap (to dipeptides), Trg (to ribose and galactose) and Aer (to oxygen). These are arranged in arrays of three receptors. Ligands can bind to many different binding sites on the portion of each MCP that extends into the periplasm outside the cytoplasmic membrane but within the outer membrane of the cell. When ligands bind to the receptors, they induce a change in conformation that is transmitted down the length of the protein to enable binding with a kinase that phosphorylates CheA. This activity in the Sensing module (shown on the left side of figure 1) begins a cascade that culminates in the phosphorylation of CheY in the Motility module.

The Sensing module, as described so far, only detects the concentration of metabolites or toxins, not whether it is increasing or decreasing. To detect that, the MCPs adapt in response to the previous concentration so that they measure a change. This is accomplished by two other Che proteins: CheR constitutively methylates the MCPs while CheB demethylates them in response to the phosphorylation of CheW [38,39]. The stronger the input signal is at a given moment, the more methylated the MCPs are at the next, requiring a still stronger input to generate an output on the next cycle.

The whole complex of receptors and Che proteins constitutes the control mechanism responsible for modulating the operation of the flagellum (the production mechanism). This is an impressive mechanism that is highly adaptive. It responds not only to gradients as concentrations vary over five orders of magnitude, but also to the intracellular energy status through the Aer receptor [48], and to the presence of other bacteria by altering the number of different receptors [38]. The number of receptors of different types also varies between individual bacteria, resulting in individual differences in making the decision to move forward or tumble.^2^ This whole mechanism is situated in an organism in which it serves, among other things, to create the conditions for constructing both the production and control mechanisms and to provide energy for their operations. These production and control mechanisms are thus constituents of an autonomous system.

## 4. Control mechanisms and decision-making

Having introduced the distinction between production and control mechanisms, we turn to the relation between control mechanisms and the cognitive activity of decision-making. Various theorists advance different criteria for attributing cognition. The dominant strategy, as exemplified in the development of both cognitive psychology and cognitive science, has been to take human beings as the reference point and to focus on distinctively human cognitive activities. In these disciplines, humans serve as their own model organisms; practitioners often explicitly reject the continuity of cognitive phenomena across all domains of life. Lyon [7] characterized this as the anthropogenic approach, which she contrasts with a biogenic approach that has roots in theorists such as Maturana [50]. This approach locates cognition in the demands that organisms must meet in order to maintain and reproduce themselves. Among those who embrace the biogenic approach, there are differences regarding how far back in phylogeny to identify cognition. Some, following Piaget [51], Maturana [6,50] and von Foerster [52], among others, identify cognition as an activity of all living organisms [53,54]. This thesis, for which Heschl [55] coined the name ‘Life = Cognition Thesis’, maintains that the dimension of a living organism interacting with its environment and modifying itself internally without losing its identity coincides with cognition [56,57]. Formulated in these terms, the thesis countenances all interactions between an organism and its environment, fails to identify the mechanisms underlying cognitive capabilities, and cannot distinguish cognition from mere causal interactions between organisms and their environments [58].

Some theorists are more restrictive, limiting cognition to organisms with rich behavioural capabilities [59]. Moreno *et al.* [60] contend that identifying cognition with life renders cognition indistinguishable from purely biological processes, making it difficult to understand the nature, function and the evolutionary history of cognition as a specific phenomenon with its own normative prescriptions. Barandiaran & Moreno [2] insist that a nervous system is required to provide the high degree of organizational complexity needed for cognition to manifest its own normativity, distinct from the metabolic norm of self-maintenance. A consequence of distinguishing cognition from the activities of all living organisms is that these authors introduce a chasm between cognition and self-maintenance. One cannot use organisms without neurons such as plants, fungi and unicellular systems as model organisms that realize simpler, and therefore more accessible, instances of cognition.

We embrace the view that cognitive activities are performed by all living organisms. Yet we identify as activities that may be considered cognitive in a minimal sense—or necessary or relevant for grounding cognition—not all possible activities performed by or within an organism, but only those activities resulting at least from the actions of control mechanisms. And we focus on what is distinctive about these activities—they involve the exercise of control over metabolic and agential activities. Norms, in this case, are those that figure in control operations. To develop this perspective, we focus on one activity that is usually considered as fundamentally cognitive—decision-making [61,62].

Decision-making occurs when, in the face of alternative modes of operation, a system detects and integrates information procured from multiple sources (*measurement*), based on this information evaluates alternative modes of operation and, depending on that *evaluation*, *selects* a mode of operation. All present-day living beings have this capacity, as illustrated in the previous section in the case of bacteria. The need for decision-making is perhaps even more apparent in eukaryotic and multicellular organisms. The specialization of organelles in eukaryotes requires control mechanisms that decide which organelles to operate at what rate [63,64] and where they should be positioned, depending on the needs of the cell [65]. A similar need for control and decisions arises as different cells specialize in performing different activities in multicellular organisms [66,67]. Neurons constitute a specialized type of cell for integrating information and determining which activities to perform, and in vertebrates, specialized neural structures such as the basal ganglia take on the function of regulating both motor activities and other neural processing [68,69].

Situating decision-making in the framework of control mechanisms allows us to identify its core features and to underline the importance of studying it in simpler model organisms. As we argued in §2, all organisms must control and coordinate production mechanisms so that they function in ways appropriate given the organism's internal state and environmental conditions. Control mechanisms enable organisms to selectively modulate their own dynamics (production mechanisms or other control mechanisms) according to their own internal processes based on what they detect about both their internal state and conditions in the environment. The response of the organism is not just a reflex as it relies on the constraints currently realized in the control mechanism but subject to adjustment, not on the production mechanisms alone. Individual constraints determine how the control mechanism categorizes sensory inputs from internal and external conditions into ‘equivalence classes’ [70]. These constraints not only group inputs into equivalence classes but evaluate them so as to select actions [58]. That is, the constraints in the control mechanism embody norms about what to do in response to an input that it has classified into an equivalence class. Moreover, the constrains that constitute the control mechanisms are not fixed. They are made by the organism itself [71] and are subject to control activities of other control mechanisms in the organism [29]. As a result, the same first-order control mechanism may realize different norms and make different decisions on different occasions.

## 5. Heterarchical organization of decision-making mechanisms

**Figure 2.**
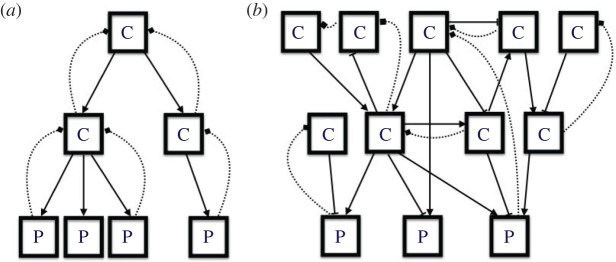
(*a*) A typical hierarchical arrangement of control mechanisms. (*b*) A heterarchical arrangement. Sensory inputs are represented by dotted arrows, control by solid arrows. Edge-ended lines indicate inhibition.

Given our characterization of an organism as embodying multiple control mechanisms, each functioning as a decision-making mechanism, an important question concerns how these multiple control/decision-making mechanisms are organized. Theorists often envisage multiple mechanisms as organized hierarchically. Simon [72] argue that natural and human-designed systems are (nearly) decomposable systems in which components are combined into larger systems, which are then combined into yet larger systems. This framework, which applies to production mechanisms, is often extended to control mechanisms. Humans organize many social systems hierarchically: in both business and the military, lower-level bosses or officers report to higher-level ones, with a president or a general ultimately in charge. The structure corresponds to a pyramid in which there are multiple low-level controllers, few controllers at higher levels and just one top-level controller (figure 2*a*) [73]. Strict hierarchy is seldom realized in human social systems, as different intermediate agents often operate on their own, and management theorists have explored a variety of ‘post-bureaucratic’ modes of social organization that often depart dramatically from hierarchical organization [74,75].

The notion of hierarchy is even less applicable to control mechanisms in living systems, where the pyramid is often inverted—with multiple control mechanisms operating on the same production mechanism. Each controller makes its own decisions about the operation of the production mechanism. This picture is iterated as control mechanisms operate on and are operated on by multiple other control mechanisms. Although Pattee [27,76] draws upon the framework of hierarchical organization in analysing control in biology, in one publication he adopts McCulloch's [15] term *heterarchical*, describing a *heterarchical network* as ‘a distributed causal network that does not define an order relation or special significance to particular local causal links' [77, p. 220]. Winning & Bechtel [9] describe how control contains a local hierarchical component (the controller operates on the controlled system), but that as one tries to move higher, the hierarchy quickly gives way to a tangled heterarchy of independently operating control mechanisms (as sketched in figure 2*b*).

Heterarchical organization of control networks in biological system is not just an observation—it is what we should expect. When humans try to establish hierarchical organization in institutions such as business and the military, they often design organizational charts that they then seek to implement and enforce. Control mechanisms in living organisms are not the product of such design; they have arisen through evolutionary processes in which variants that improve, or at least do not seriously diminish, the ability of organisms to maintain themselves and leave offspring become fixed as a result of selection or drift. In such an evolutionary process, one should expect new control mechanisms to be added independently of one another in an opportunistic manner.

Insight into the nature of this process can be gleaned from the way in which designers often confront problems with their machines or their computer code—they make changes, known popularly as *kluges* or *kludges* [78], that are intended specifically to solve the immediate problem. These often are not elegant but are sufficient. Although sometimes treated as shortcuts to proper design, kludges often represent sound strategies; when one has an otherwise functioning system, adding a kludge is less likely to result in serious failure than would developing a new design de novo. The art of developing a kludge is to act locally and affect as few other components as possible. This applies as well to evolution: adding a limited control mechanism to enhance fitness avoids seriously disrupting the existing set of mechanisms. Marcus [79] argues that many features of human cognition, often taken to be failures, represent kludges added to earlier evolved nervous systems. Jacob [80] made much the same point, referring to evolution as tinkering. Since there is no emphasis on maintaining the hierarchy, tinkering, kludging or piecemeal introduction of new control mechanisms is more likely to result in heterarchical organizations.

Although new control mechanisms in living organisms typically operate locally, there are instances of new control mechanisms that do have broad effects on organisms. How could they evolve? One context is when they provide coordination between independently operating production mechanisms. We offer three examples. The first is quorum sensing in bacteria. There are activities in which bacteria engage that only succeed when individuals coordinate their behaviour. Quorum sensing provides a way for individual bacteria to regulate their activities depending on the number or state of other bacteria, whether of the same species or of relevant other species, available nearby for collaborative activities [81]. This involves individual bacteria synthesizing and releasing a molecule, an *autoinducer*, into the environment and then regulating its own gene expression in response to the concentration registered by its own receptors. Schultz *et al.* [82] describe how *Bacillus subtilis* uses such an assessment in making the decision between maintaining competence and entering into sporulation. Conceptually speaking, the basic mechanism involves two processes that are already performed in many bacteria—synthesizing a molecule for release into the environment and measuring concentrations of molecules in the environment. All that is then needed is to couple these processes and to regulate the expression of genes needed for the activity in response to the measurement. Once such a mechanism is in place to control the activities of individual bacteria, it can then be used for multiple ends—for example, for pack hunting, rippling behaviour and fruiting body formation in the case of *Mxyococcus xanthus* [83,84].

An invertebrate example is the use of a network of neurons to coordinate contractile tissues in the bell of cnidarians. Drawing on Holland's [85] characterization of this network as a *skin-brain*, Keijzer *et al.* [86] advanced the skin-brain thesis according to which a first role of neurons was to cause contractile cells to contract together (e.g. in the bell, where coordinated contraction enables swimming). The cnidarian nerve net is the recipient of information from multiple sources that it must integrate to make decisions about motor behaviours [87]. As a third example, central pattern generators (perhaps better called *local pattern generators* since they are local to individual muscle systems) function similarly to coordinate the contraction of muscles in most animals [88]. This pattern of using neural connections to coordinate local systems is further illustrated in the nervous system of vertebrates, in which neurons in the reticulospinal system [89] and the mesencephalic locomotor region [90] serve to coordinate the activity of multiple pattern generators, enabling coordinated limb movement. Even in organisms with a greatly expanded forebrain, which allows for the integration of vastly expanded sensory information in decisions about actions, these local integratory systems themselves make decisions required for action [91].

Phrased in terms of decision-making, what heterarchy entails is that decision-making is highly distributed in living organisms. Rather than a central system making all the major decisions, leaving subordinate decision-makers only to make decisions required in the course of implementation, there are multiple decision-makers operating relatively independently of each other. Integration often occurs at lower levels, at which control mechanisms may receive conflicting outputs from various decision-makers and have to integrate them into one action. Cognitive science research has tended to focus only on very high-level, explicit, decisions which might be presented to a human participant in a controlled experiment [92,93]. This ignores the substantial number of decisions made within participants, including ones affecting behaviour in the experimental circumstances as well as ones made in the daily lives of the participants [69].

The heterarchical nature of control will be furthered illustrated in the next section, in which we focus on another feature of decision-making—the reliance on internal models of external phenomena. This is often thought to require a nervous system, but we will illustrate it with an example from bacteria.

## 6. Basing decisions on an internal model of environmental time

So far we have argued for viewing control mechanisms in a wide range of organisms as making decisions and hence as cognitive. As we noted above, some theorists, such as Barandiaran & Moreno [2], argue for withholding the language of cognition from processes directly integrated with production mechanisms. They argue that only when a control mechanism uses neurons can it employ norms distinct from those involved in metabolism and so count as cognitive. One way neurons seem to allow for distinct norms is by enabling organisms to develop internal models of the world and acting to act on those. In this section, we show that even this is possible without neurons by showing how cyanobacteria are able to internally model time in their environment and use this to control their metabolic and other activities.

An important feature of the environment that affects most organisms on Earth is the light–dark cycle. It determines when plants and cyanobacteria can carry out photosynthesis or when it is safe for mammals to move about in their environment. For all organisms, periods of sunlight increase risks of DNA damage due to UV exposure and to elevated ambient temperature in which they must function. Coping with this cycle was particularly important for cyanobacteria, which evolved production mechanisms that enabled them to procure energy through photosynthesis. Molecular oxygen, a byproduct of photosynthesis, is toxic for many enzymes, including nitrogenase, which the bacteria need to fix nitrogen [94]. Some species of cyanobacteria solved this problem by differentiating into specialized cell types, one of which fixes nitrogen and the other of which performs photosynthesis [95]. These cells must then coordinate their activities so that each can meet the needs of the other. As first observed by Stal & Krumbein [96], other species of cyanobacteria segregate photosynthesis and nitrogen fixation in time. After raising bacteria from the genus *Oscillatoria* on a 16 h L : 8 h D cycle, these researchers observed that nitrogenase activity increased and photosynthesis ceased just before the onset of darkness. The bacteria maintained this cycling even when transferred to constant light.

Although Stal & Krumbein did not characterize this ability as circadian, researchers studying both plants and animals had much earlier demonstrated endogenously generated rhythms of approximately 24 h and established that they regulated a variety of behaviours such as folding of leaves in some species of plants [97] and locomotion in animals [98]. Halberg [99] coined the term *circadian* (*circa*, about + *dies*, day) for these rhythms and researchers determined that they involved not just an endogenously generated rhythm of about 24 h but also a period that is not affected by ambient temperature (temperature-compensated, unusual for chemical reactions) and a phase that can be entrained to the light–dark cycle [100]. Working with a freshwater cyanobacterium of the genus *Cyanothece*, Grobbelaar *et al.* [101] established that the switch between photosynthesis and nitrogen fixation met the first criterion: if raised in constant light, the bacterium fixed nitrogen at any time, but after being switched to a light–dark cycle, it would confine nitrogen fixation to the dark phase even after it was switched back to continuous light. Subsequently, Chen *et al.* [102] established that the rhythms were temperature-compensated and entrainable by exposure to dark or to a low temperature (0°C) pulse.

**Figure 3.**
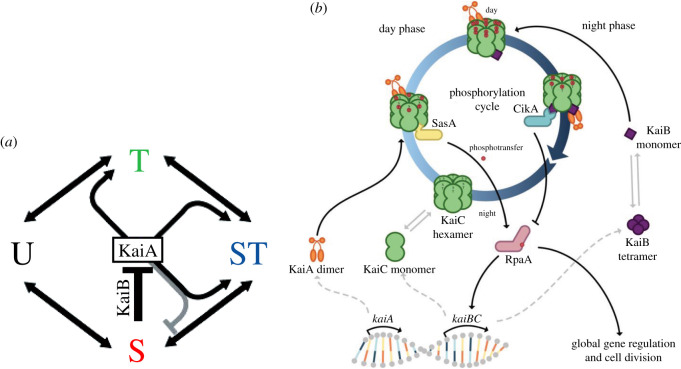
(*a*) The sequence of phosphorylation states constituting the circadian clock in cyanobacteria. From [105]. Reprinted with permission from AAAS. (*b*) The cyanobacterial circadian clock. See text for details. Figure reprinted from [106] under Creative Commons License (CC BY-SA 4.0). (Online version in colour.)

The ability of cyanobacteria and other organisms to maintain an internal model of the light–dark cycle of their environment posed a challenge: what kind of mechanism could track daily time? Ishiura *et al.* [103] identified three genes (which they named *kaiA*, *kaiB* and *kaiC* after the Japanese word *kaiten*, ‘turning of the heavens') that were necessary for circadian rhythms in the cyanobacterium *Synechoccus elongatus*. Nakajima *et al.* [104] reported circadian rhythms in a preparation involving just ATP and the three Kai proteins, suggesting a cycle of phosphorylation and dephosphorylation. Specifically, they proposed that two sites on KaiC—serine residue 431 (S) and threonine 432 (T)—were phosphorylated and then dephosphorylated every 24 h. Rust *et al.* [105] established that these phosphorylations occur in a specific sequence—T is the first site phosphorylated, followed by S, and then T is the first site dephosphorylated, followed by S (figure 3*a*). The four different phosphorylation states corresponded to different times of day—U (both unphosphorylated) to early morning, T only to late afternoon, both T and S to early evening, and S only to late night. To ensure that the process proceeded in just one direction, Rust *et al.* [105] proposed that KaiA promoted both the phosphorylation operations and, once phosphorylated, KaiB would serve to inhibit KaiA and allow KaiC to dephosphorylate itself. The state of phosphorylation of KaiC therefore serves as an internal model of time of day [107].

Over the following decade, a fuller understanding emerged [108]. KaiC usually exists as a hexamer with two domains (often represented, as in figure 3*b*, as a double doughnut). When KaiC is unphosphorylated, KaiA binds to the tails that extend out from the CII domain (shown on the left side of the cycle), altering the conformation of KaiC and allowing phosphorylation. Phosphorylation further alters the conformation, affecting the CI domain so that KaiB can now bind [109]. As the conformation of KaiC changes, the tails to which KaiA binds are withdrawn and KaiA binds instead to KaiB (shown on the right side of the cycle).

For this mechanism to satisfy our account of a control mechanism, two further requirements must be met. First, it must actually track the light–dark cycles in its environment. This requires some means by which the oscillator can be entrained to that cycle. Since *Synechococcus elongatus* has no photoreceptors, it uses proxies to detect the light–dark cycle. For example, when the bacterium is engaged in photosynthesis, quinones are in a reduced state. With darkness they oxidize rapidly, bind to both CikA and KaiA, and cause them to degrade [110]. By delaying phosphorylation of KaiC, the metabolic state that results when photosynthesis ceases with darkness acts to reset the clock. Second, the circadian mechanism must act in some manner on the production mechanism of gene expression. When SasA is bound to KaiC, it phosphorylates itself and transfers this phosphate to RpaA (shown below the cycle in figure 3*b*) [108,111]. Phosphorylated RpaA acts as a transcription factor, binding to approximately 100 Class 1 promoters, upregulating expression of approximately 170 genes (including *kaiB and kaiC*). Eight of these genes are also transcription factors which upregulate yet other genes. Phosphorylated RpaA also acts to downregulate genes with Class 2 promoters. When KaiB binds to KaiC, it dislodges SasA, stopping the phosphorylation of RpaA. When KaiA joins KaiB, CikA also binds to and dephosphorylates RpaA. Together these actions remove the activation of Class 1 promoters and the repression of Class 2 promoters. With repression removed, genes controlled by Class 2 promoters begin to be transcribed.

Employing this mechanism *S*. *elongatus* is able to use an internal model of the light–dark cycle in its environment to make decisions about which genes to express [107]. It satisfies the requirement of a control mechanism in acting on flexible constraints in the mechanism of gene expression. Through the entrainment process, the clock maintains a connection to the day–night cycle in the environment, but this only indirectly affects its decision-making, which is based locally on the state of the clock itself. When the entrainment process is inhibited, e.g. by keeping the bacteria in constant light, the lineage of descendants maintains circadian gene expression for two weeks (the longest period that was measured) [112].

One function for which neurons are often thought to be required is to maintain internal models of the environment when sensory cues are not available. They often do so, but the cyanobacterial clock shows that this is not required; the molecular mechanisms in the clock establish norms that regulate the bacterium's behaviour. Interestingly, although neurons do play a role in animal circadian clocks, the core mechanism is also an intracellular chemical process [113,114]. This conforms to one of Sterling & Laughlin's [115] principles of neural design: compute intracellularly and chemically whenever possible. Before leaving this example, though, we should emphasize that circadian control is only one of several control mechanisms that determine gene expression. Gene expression is also controlled by a host of other control mechanisms that adapt to such factors as available nutrients and toxins. The circadian clock is just one component in a heterarchy of decision-making processes.

## 7. Conclusion

By advancing an account of control mechanisms as operative on production mechanisms in biological autonomous systems, we have shown that control mechanisms are organized heterarchically and that they make decisions, an activity commonly associated with cognition.

A major advantage of this account is the fact that control mechanisms are ubiquitous in biological systems, thus radically expanding the range of model organisms in which we can study decision-making. In particular, it allows us to focus on relatively simple organisms such as bacteria, which are potent models to identify and analyse the core elements of a cognitive process such as decision-making and the control mechanisms responsible for it, in line with the general approach developed within this theme issue and with a long tradition of research that emphasizes the continuity between biological and cognitive phenomena [1]. In the case of decision-making, the core elements are: (1) selecting between alternatives based on making measurements of both internal and external conditions and (2) evaluating those by means of norms embodied in the control mechanism, which are ultimately rooted in the maintenance of the organism. We have illustrated these basic elements of decision-making in bacteria, and showed how they apply also in multicellular systems. On the basis of these examples, we have argued that control mechanisms are organized heterarchically and that even in minimal cases they can use internal models of the world to make decisions.

The question of whether cognition coincides with life or, if not, where it starts, might remain open to discussion. Bacteria do not perform all the cognitive activities other organisms perform. Some organisms make decisions in ways that go beyond the capacities of bacteria, for example by projecting possible outcomes of possible actions during evaluations. This requires more complex control mechanisms than found in bacteria and other relatively simple organisms. These differences, however, do not undermine the importance of such model organisms in identifying and characterizing the distinctive features of control mechanisms and of their activity of decision-making and, importantly, in understanding control in terms of heterarchy instead of hierarchy. Adopting this theoretical framework developed from minimal cases may be fruitful for understanding decision-making and the architecture of control in parts of the nervous system of vertebrates, such as the brainstem, hypothalamus and basal ganglia, which do not realize a centralized or pyramidal architecture and which are part of a control network that includes many other parts of the nervous system and of the whole organism, exhibiting ways of operation that are similar in significant ways to those we have identified in simpler organisms [69,116].

## Endnotes

1 The autonomy framework provides a similar distinction between the first-order constitutive constraints acting on processes and higher-order regulatory constraints acting on lower-order ones [11]. An analysis of the convergences and differences between the two accounts is beyond the scope of this paper, where we will adopt the mechanist terminology.

2 In unicellular systems, sensory modules can achieve a very high degree of structural sophistication and precision. One example is the eukaryotic unicellular dinoflagellate *Erythropsidinium*. It exhibits a light-sensitive camera-type ocular organelle (with retina-like photoreceptors element and refractive lens-like structures) of multiple endosymbiotic origin, capable of changing its morphology in different light conditions and efficiently exploiting weak light in deep water to orient movement [49].

## Data accessibility

This article has no additional data.
